# Flavones inhibit breast cancer proliferation through the Akt/FOXO3a signaling pathway

**DOI:** 10.1186/s12885-015-1965-7

**Published:** 2015-12-16

**Authors:** Chia-Hung Lin, Ching-Yao Chang, Kuan-Rong Lee, Hui-Ju Lin, Ter-Hsin Chen, Lei Wan

**Affiliations:** 1 Institute of Molecular Medicine, National Tsing Hua University, No. 101, Section 2, Kuang-Fu Road, Hsinchu, 30013 Taiwan; 2 Department of Biotechnology, Asia University, Taichung, Taiwan; 3 Department of Ophthalmology, China Medical University Hospital, Taichung, Taiwan; 4 School of Chinese Medicine, China Medical University, No. 91, Hsueh-Shih Road, Taichung, 40402 Taiwan; 5 Graduate Institute of Veterinary Pathobiology, College of Veterinary Medicine, National Chung Hsing University, Taichung, Taiwan; 6 Department of Gynecology, China Medical University Hospital, Taichung, Taiwan

**Keywords:** Breast cancer, Flavones, FOXO3a, Akt, p27, Apoptosis

## Abstract

**Background:**

Flavones found in plants display various biological activities, including anti-allergic, anti-viral, anti-inflammatory, anti-oxidation, and anti-tumor effects. In this study, we investigated the anti-tumor effects of flavone, apigenin and luteolin on human breast cancer cells.

**Methods:**

The anti-cancer activity of flavone, apigenin and luteolin was investigated using the MTS assay. Apoptosis was analyzed by Hoechst 33342 staining, flow cytometry and western blot. Cell migration was determined using the culture inserts and xCELLigence real-time cell analyzer instrument equipped with a CIM-plate 16. Real-time quantitative PCR and western blot were used to determine the signaling pathway elicited by flavone, apigenin and luteolin.

**Results:**

Flavone, apigenin and luteolin showed potent inhibitory effects on the proliferation of Hs578T, MDA-MB-231 and MCF-7 breast cancer cells in a concentration and time-dependent manner. The ability of flavone, apigenin and luteolin to inhibit the growth of breast cancer cells through apoptosis was confirmed by Hoechst33342 staining and the induction of sub-G1 phase of the cell cycle. Flavone, apigenin and luteolin induced forkhead box O3 (FOXO3a) expression by inhibiting Phosphoinositide 3-kinase (PI3K) and protein kinase B (PKB)/Akt. This subsequently elevated the expression of FOXO3a target genes, including the Cyclin-dependent kinase inhibitors p21^Cip1^ (p21) and p27^kip1^ (p27), which increased the levels of activated poly(ADP) polymerase (PARP) and cytochrome *c*.

**Conclusion:**

Taken together, these data demonstrated that flavone, apigenin and luteolin induced cell cycle arrest and apoptosis in breast cancer cells through inhibiting PI3K/Akt activation and increasing FOXO3a activation, which suggest that flavone, apigenin and luteolin will be the potential leads for the preventing and treating of breast cancer.

## Background

Breast cancer is one of the most common types of cancer affecting women in western countries, and in recent years, the number of deaths caused by breast cancer has been increasing in Taiwan. Despite the new promising breakthrough in therapeutics, the annual breast cancer mortality rate continues to increase, and one million new cases are diagnosed every year [1]. Numerous risk factors for breast cancer etiology have been identified, including genetic, gender, age, alcohol consumption, smoking and obesity [2–5].

Breast cancer develops as a consequence of cellular changes that increase the rate of cell division and metastasis, decrease the rate of apoptosis, or both. These changes often involve dysregulation of key signal transduction pathways within the cell that transmit extracellular signals to transcription factors, resulting in changes in gene expression. Previous studies have shown that increased protein kinase B (PKB)/Akt activity can promote breast cancer cell survival and therapeutic resistance [6, 7]. Forkhead box O3 (FOXO3a), a downstream target of the phosphatidylinositol-3-kinase (PI3K)/Akt pathway, belongs to a family of transcription factors that are characterized by a distinct forkhead DNA-binding domain [8]. Activation of PI3K/Akt signaling causes phosphorylation of FOXO3a, thereby inhibiting its activity and translocating it out of the nucleus, where it becomes subject to proteasomal degradation in the cytosol [9]. The cytoplasmic expression level of FOXO3a is correlated with Akt phosphorylation and is associated with poor prognosis in breast cancer [10]. Nuclear localization of FOXO3a promotes the expression of multiple target genes such as p21^Cip1^ (p21), p27^kip1^ (p27), and cyclin D, which results in cell cycle arrest to inhibit the growth of cancer cells [9, 11, 12]. Cell cycle arrest in the G1, G2-M, and S phases can lead to apoptosis [13, 14]. FOXO transcription factors are also involved in the cellular stress response, and they regulate cell cycle progression and apoptosis [15]. In particular, FOXO3a plays a vital role in the initiation of cell cycle arrest, in addition to its involvement in DNA damage-mediated apoptosis [16]. Moreover, FOXO3a is an important tumor suppressor that is underexpressed in many breast cancers. Several anti-cancer drugs have been shown to increase the expression of FOXO3a, which suggest it is a tangible therapeutic target for breast cancer therapy [17].

Surgical resection, radiation therapy, and chemotherapy are among the main treatment options for breast cancer patient. In addition, there is growing need to discover new chemopreventive agents that are effective in preventing/treating breast cancer. Polyphenols are compounds found in food plants and Chinese herbs, and they can be divided into various classes on the basis of their molecular structure. Flavonoids constitute a class of polyphenols that can be further divided into the following six subclasses: flavones, flavanones, flavanols, flavonols, flavonols, isoflavones, and anthocyanidins [18, 19]. A recent study showed that flavonoids exhibit various beneficial biological properties such as anti-inflammatory [20, 21], anti-viral [22, 23], anti-allergic [24], anti-oxidant, [25, 26] and anti-tumor activities [27–31]. Researchers have also discovered that flavones inhibit tumor growth by promoting apoptosis in cancer cells [27, 28, 32]. Compounds in the flavone subclass, including flavone, apigenin, and luteolin, are present in fruits and vegetables and are considered to be potent dietary phytochemicals that are effective in cancer chemoprevention [33, 34].

Although different mechanisms and signaling pathways have been proposed as targets of flavone, apigenin, and luteolin, these compounds were studied individually and occasionally by using different model cancer cells. However, whether these structurally similar compounds could induce common pathways among different types of cancer cells to potentiate their chemoprevention activity remains to be elucidated. The objectives of the present study were to investigate the anti-proliferative role of flavone, apigenin, and luteolin in MCF-7, Hs578T, and MDA-MB-231 human breast cancer cells, and to elucidate the common molecular pathways in these cells.

## Methods

### Cell culture

The human Breast cancer cell lines Hs578T, MDA-MB-231 and MCF-7 were purchased from American Type Culture Collection. Cells were cultured in Dulbecco’s modified Eagle medium (Invitrogen), supplemented with 10 % fetal bovine serum (Invitrogen) and 1 % penicillin-streptomycin solution (Invitrogen) at 37 °C in a 5 % CO_2_ incubator. No approvals were required for this study, which complied with all relevant regulations.

### Cell viability assay

Cell viability was determined using the MTS/PMS ((3-(4,5-dimethylthiazol-2-yl)-5-(3-carboxymethoxyphenyl)-2(4-sulfophenyl)-2H-tetrazolium, inner salt)/phenazine methosulfate) assay. Hs578T (2 × 10^3^ cells/well), MDA-MB-231 (2 × 10^3^ cells/well) and MCF-7 (4 × 10^3^ cells/well) cells were seeded in 96-well plates. Media containing different concentrations (0–100 μM) of the flavone (HPLC > 98 %, Sigma), apigenin (HPLC > 95 %, Sigma) and luteolin (TLC > 98 %, Sigma) were added and incubated for 72 h. Subsequently, 20 μl of MTS was added from a stock solution (2 mg/mL) and incubated for an additional 2 h. The absorbance was read at 490 nm in the microplate reader 550 model (Bio-rad).

### Hoechst 33342 staining for detection of cell apoptosis

Apoptosis was analyzed by Hoechst 33342 staining. The MCF-7, Hs578T, and MDA-MB-231 cells were seeded in 6-well plates (2 × 10^5^ cells/well) and treated with IC_50_ concentrations (Table 1) of flavone, apigenin and luteolin for 24 h. Cells were stained with 40 mg/mL Hoechst 33342 (Sigma). Nuclear morphology was assessed using the cell membrane penetration DNA dye Hoechst 33342. Then, the cells were visualized under a fluorescence microscope with a blue filter.

**Table 1:**
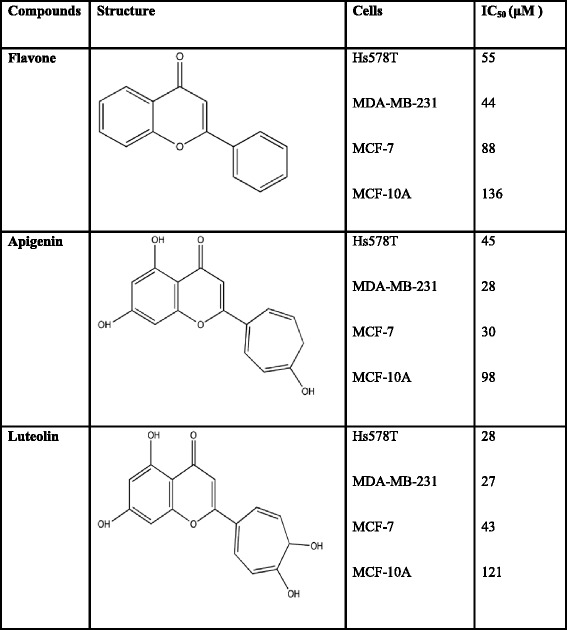
Chemical structures and IC_50_ values in flavone, apigenin and luteolin on Hs578T, MDA-MB-231, and MCF-7 breast cancer cells

### Cell cycle analysis

For cell cycle analysis, cells were seeded in a 10-cm dish (5 × 10^5^ cells) and allowed to adhere overnight. Cells were then treated with IC_50_ concentrations (Table 1) of flavone, apigenin and luteolin for 24 h. Cells were harvested and fixed in ice-cold 70 % ethanol overnight at −20 °C. Cells were washed with ice-cold PBS and subsequently suspended in staining buffer (20 μg/mL propidium iodide; 0.1 % Tween 20; 0.2 mg/mL RNase A in PBS), and incubated at room temperature for 30 min. Using flow cytometry (FACSort instrument and analysis software; ModFit LT), the cells were analyzed with regard to cell cycle distribution and apoptosis.

### Colony formation assay

Cells were plated in a 25 T flask at a density of 2 × 10^3^ cells and treated with IC_50_ concentrations (Table 1) of flavone, apigenin and luteolin for 21 d. Thereafter, the cells were then fixed with methanol for 15 min, followed by incubation with 0.5 % crystal violet for 30 min and rinsing with deionized distilled water.

### Wound healing assay

Cell migration was determined using the culture inserts (ibidi). Cells were then trypsinized, counted, plated into each well of the culture-inserts (3.5 × 10^4^ cells per well), and incubated at 37 °C in a humidified atmosphere with 5 % CO_2_. Cells were allowed to attach for 12 h, and then the culture inserts were gently removed. MCF-7 breast cancer cells were treated with flavone (88 μM), apigenin (30 μM), and luteolin (43 μM) for 24 h. Images were processed and analyzed using Image Plus software.

### Analysis of cell migration and invasion

To monitor cell migration/invasion in real time, we used the xCELLigence Real-Time Cell Analyzer (RTCA) DP Instrument equipped with a CIM-plate 16 (Roche, Indianapolis, IN), which is a 16-well system in which each well is composed of upper and lower chambers separated by an 8-μm microporous membrane. Migration/invasion was measured as the relative impedance change (cell index) across microelectronic sensors integrated into the bottom side of the membrane. For cell migration experiments, cells (7.5 × 10^4^ /well) were added in duplicates to the upper chambers. MCF-7 breast cancer cells were treated with flavone (88 μM), apigenin (30 μM) and luteolin (43 μM). Migration/invasion was monitored every hour for 9 h. For quantification, the cell index at the indicated time points was averaged from three independent measurements.

### RNA extraction, reverse transcription (RT), and real-time PCR

Total RNA from Hs578T, MDA-MB-231 and MCF-7 cells was extracted using the RNeasy Mini Kit (Qiagen, Valencia, CA). RNA (4 μg) was reverse-transcribed using the Superscript First Strand synthesis system for conversion to cDNA (Invitrogen, Carlsbad, CA). Primers and probes for amplification and detection were selected from the Universal Probes Library (Roche, UK). The primer sequences used were as follows: *FOXO3a* (forward: acaatagcaacaagtataccaagagc, reverse: gactgtcgtcagctgattcg), *p21* (forward: gcgactgtgatgcgctaat, reverse: tcgaagttccatcgctcac), and *p27* (forward: ccctagagggcaagtacgagt, reverse: agtagaactcgggcaagctg). Amplification was performed in a LightCycler480 system (Roche, UK) beginning with an initial heating at 95 °C for 10 min, followed by 40 cycles of 95 °C for 15 s, 60 °C for 10 s, and 72 °C for 1 s. Gene expression levels were determined using glyceraldehyde 3-phosphate dehydrogenase as a control.

### Western blot

Whole cell lysates were obtained by direct lysis of the cells using an ice-cold Mammalian Protein Extraction Reagent (M-PER, Pierce). Nuclear and cytoplasmic fractionations were performed using the Nuclear and Cytoplasmic Extraction Kit (Pierce). Protein (20 μg) was resolved by 10 % SDS-PAGE and electro-transferred onto a polyvinylidene difluoride membrane. Western blotting was performed according to standard methods, using anti-cleaved-PARP, anti-p53, anti-cytochrome *c*, anti-Akt, anti-phosphorylated-Akt (ser473), anti-FOXO3a, anti-p21, anti-p27 and anti-β-actin antibodies (Cell Signaling Technology). The membranes were developed using an enhanced chemiluminescence detection system, horseradish peroxidase substrate (Millipore) and an ImageQuant LAS-4000 Chemiluminescence and Fluorescence Imaging System (FujiFilm).

### Statistical analysis

For each study group, data were derived from at least three independent experiments. Statistical analysis was performed using a Student’s *t*-test or Chi-Square test to compare differences in values between the control and experimental groups. The results are presented as the mean ± SD. *P* < 0.05 indicated a statistically significant difference.

## Results

### Effects of flavone, apigenin and luteolin on cell viability and apoptosis of breast cancer cells

**Fig. 1:**
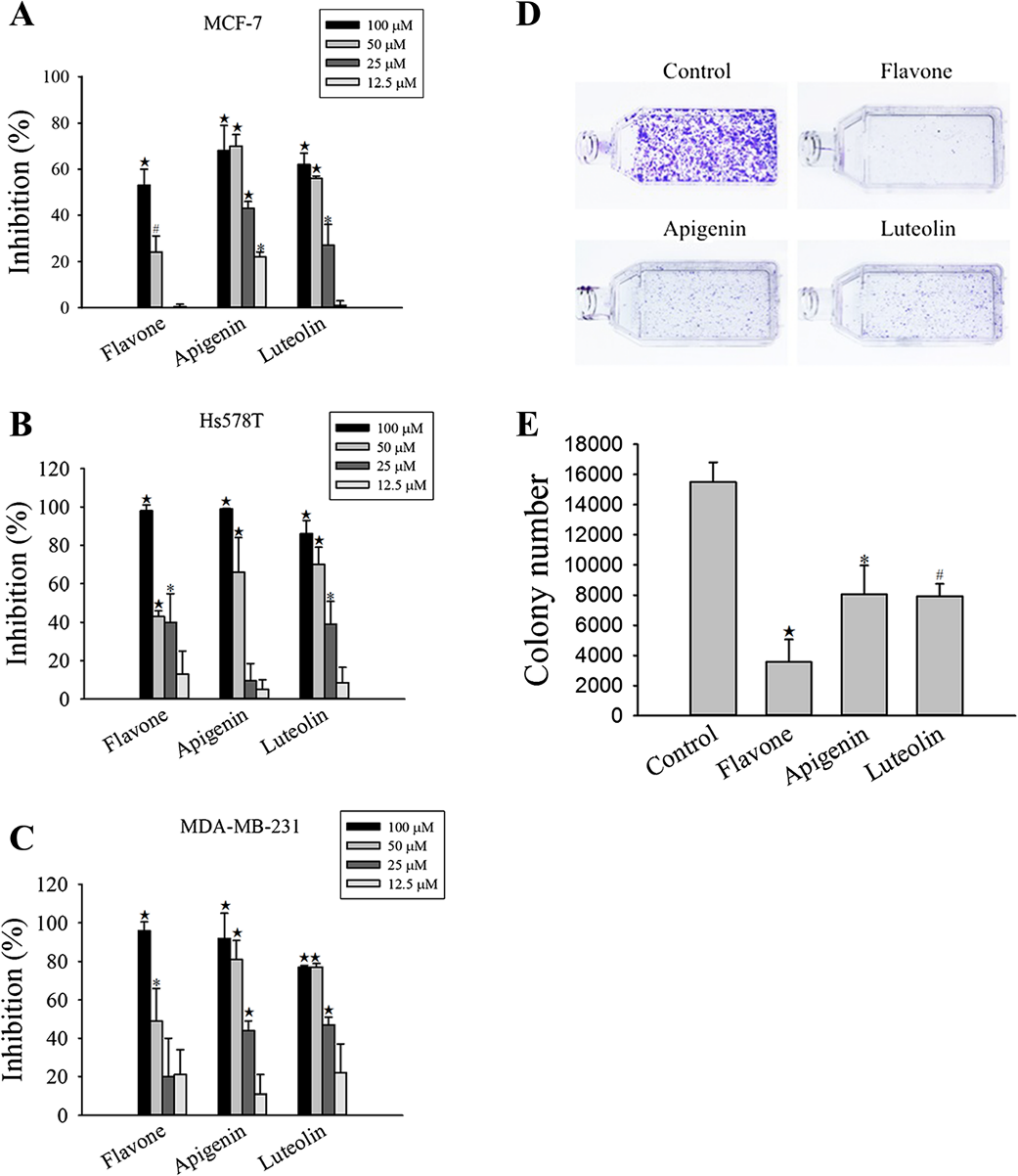
Effects of flavone, apigenin and luteolin on cell viability of MCF-7, Hs578T and MDA-MB-231 breast cancer cells. **a** MCF-7, **b** Hs578T, and **c** MDA-MB-231 cells were cultured in 96-well plates and treated with varying concentration of flavone, apigenin and luteolin (12.5–100 μM) for 72 h as indicated. Cell viability was assessed with a MTS/PMS ((3-(4,5-dimethylthiazol-2-yl)-5-(3-carboxymethoxyphenyl)-2(4-sulfophenyl)-2H-tetrazolium, inner salt)/phenazine methosulfate) assay. **d** Effects of treatment with IC_50_ concentrations (Table 1) of flavone, apigenin, and luteolin on for 21 d on colony formation in MCF-7 cells. **e** Quantification of colony numbers from colony-forming assays of untereated MCF-7 cells (control) and cells treated with flavone, apigenin, and luteolin. Results are the mean ± standard deviation of three independent experiments. *P* < 0.05 is considered as statistically significant. Symbols: *: *P* < 0.05; #: *P* < 0.01; ★: *P* < 0.001

To determine the effect of the flavone, apigenin, and luteolin on MCF-7, Hs578T, MDA-MB-231 cancer cells and on a non-tumorigenic MCF-10A cell, a MTS/PMS assay was performed. The IC_50_ for flavone, apigenin and luteolin on these cells are listed in Table 1. Flavone, apigenin and luteolin exhibited lower cytotoxic effect on MCF-10A cells than MCF-7, Hs578T and MDA-MB-231 cells (Table 1). Flavone, apigenin, and luteolin showed a concentration-dependent effect on the cell viability of MCF-7, Hs578T, and MDA-MB-231 cells (Fig. 1a, b, and c). Cell viabilities were significantly decreased treating with 12.5–100 μM flavone, apigenin, and luteolin for 72 h. Long-term effects of flavone, apigenin, and luteolin on the growth of breast cancer cells, were further assessed by colony formation assay. After 21 d of treatment with flavone, apigenin, and luteolin the colony numbers were suppressed by 2- to 3-fold (Fig. 1d, e).

**Fig. 2:**
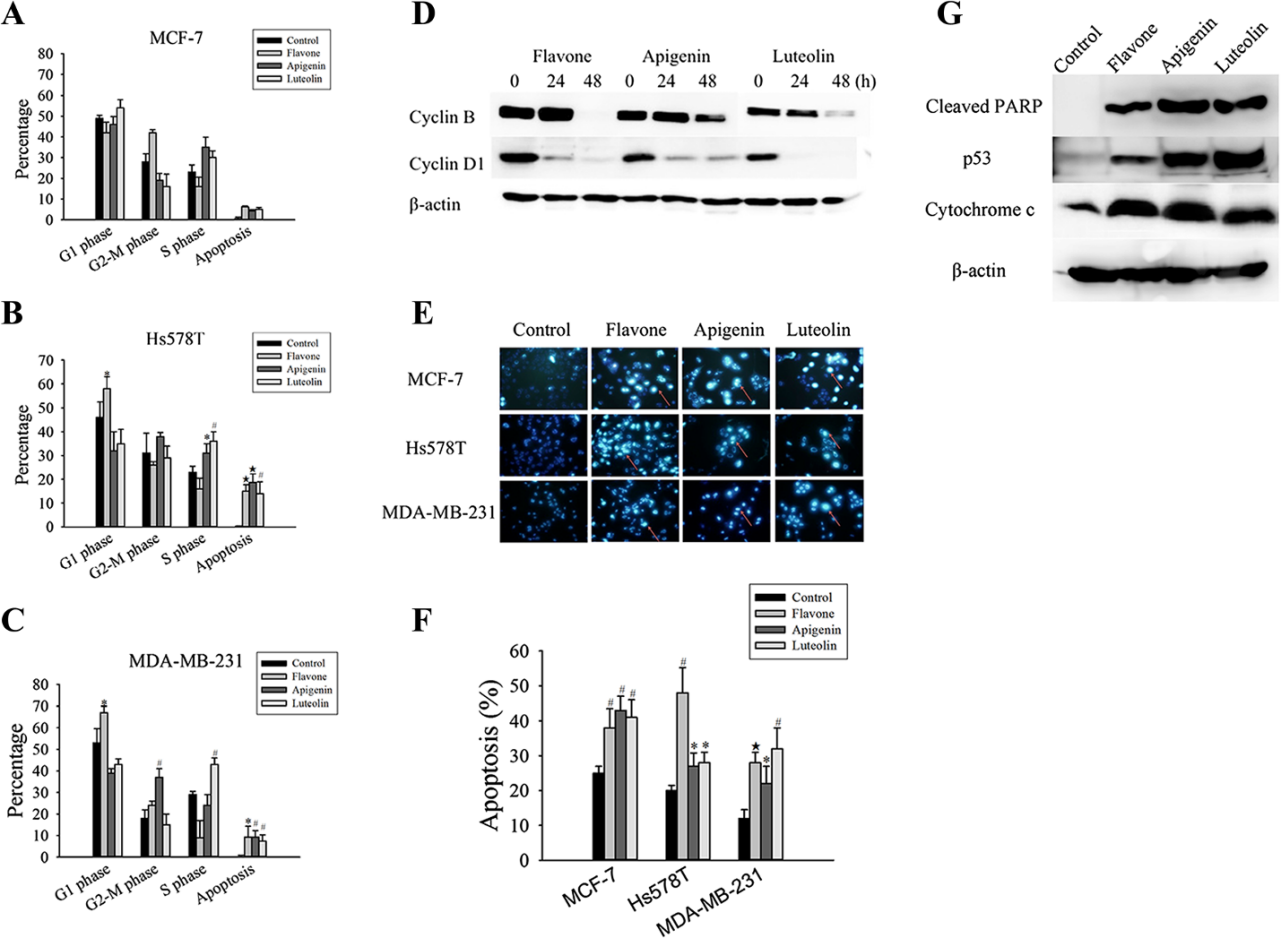
Flavone, apigenin, and luteolin induced cell cycle arrest and apoptosis in human breast cancer cells. **a** MCF-7, **b** Hs578T, and **c** MDA-MB-231 cells were treated with the IC_50_ concentrations (Table 1) of flavone, apigenin, and luteolin for 24 h prior to cell cycle analysis by propidium iodide staining. The percentage of cells in each phase of the cell cycle (sub G1, G0/G1, G2/-M and S) is indicated. **d** The effects of flavone, apigenin, and luteolin on cyclin B and cyclin D1 protein expression. Western blot analyses were performed on cell lysates form MCF-7 cells treated with the IC_50_ concentrations (Table 1) of flavone, apigenin, and luteolin for 24 h. **e** Flavone, apigenin, and luteolin induced apoptosis in MCF-7, Hs578T and MDA-MB-231 breast cancer cells as detected by Hoechst 33342 staining. The breast cancer cells were treated with the IC_50_ concentrations (Table 1) of flavone, apigenin. and luteolin. **f** Quantification of Hoechst 33342 staining of untreated MCF-7, Hs578T and MDA-MB-231 cells (control) and cells treated with flavone, apigenin, and luteolin. **g** Western blot analyses for cleaved-PARP, tumor p53, and cytochrome *c* in MCF-7 cells treated with the IC_50_ concentrations (Table 1) of the flavone, apigenin and luteolin from 24 h. Results are the mean ± standard deviation of three independent experiments. *P* < 0.05 is considered as statistically significant. Symbols: *: *P* < 0.05; #: *P* < 0.01; ★: *P* < 0.001

To delineate the mechanisms of action of flavone, apigenin and luteolin in breast cancer cells. Cell cycle analyses were performed using flow cytometry (Fig. 2a, b, and c). Flavone induced arrest at G1 phase in Hs578T and MDA-MB-231 cells, and at the G2-M phase in MCF-7 cells. Treatment with apigenin and luteolin induced arrest at G2-M and S phases in Hs578T and MDA-MB-231 cells and at S phase in MCF-7 cells. Thus, although all three compounds inhibited the growth of Hs578T, MDA-MB-231, and MCF-7 cells, their regulatory effects on the cell cycle differed.

We further examined the cyclin B, and cyclin D1 markers for G1, G2-M, and S phase arrest in the MCF-7 cells. Consistent with the observation that the flavone, apigenin, and luteolin arrested MCF-7 cell cycle at the G2-M or S phase, the expression levels of cyclin B and cyclin D1 were reduced after treatment with these compounds for 24 and 48 h (Fig. 2d).

The induction of apoptosis by flavone, apigenin, and luteolin was detected by Hoechst 33342 staining (Fig. 2e, f). Treatment with flavone, apigenin and luteolin increased the number of apoptotic cells in MCF-7, Hs578T, and MDA-MB-231 breast cancer cells. In addition, western blot analysis in MCF-7 cells revealed that the expression of p53 increased and that PARP was cleaved to its intermediate forms (Fig. 2g). The activation of PARP indicated an induction of the intrinsic apoptosis pathway by the flavone, apigenin and luteolin. Treatment with these compounds also increased the release of cytochrome *c* into the cytosol of MCF-7 cells (Fig. 2g).

### Flavone, apigenin and luteolin inhibited cell motility

**Fig. 3:**
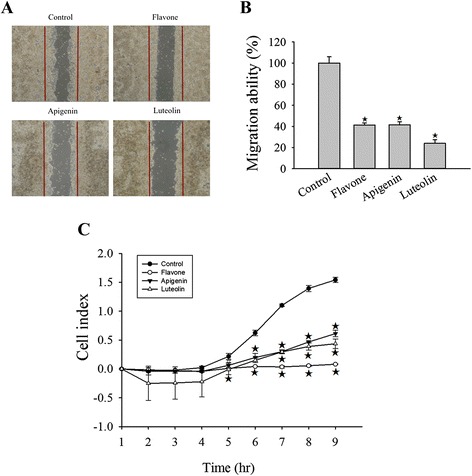
Flavone, apigenin and luteolin inhibited cell motility. **a** Representative images showing wound healing assays for cells treated with flavone (88 μM), apigenin (30 μM) or luteolin (43 μM) and an untreated control for 24 h. **b** Average number of cells that had migrated after 24 h. **c** Effects of the flavone, apigenin, and luteolin on MCF-7 cells migration. MCF-7 cells were treated with the IC_50_ concentrations (Table 1) of flavone, apigenin, and luteolin, and the real-time migration of the cells was measured using an xCELLigence system. The value of the open area at 0 h is 100 %. Results are the mean ± standard deviation of three independent experiments. *P* < 0.05 is considered as statistically significant. Symbols: *: *P* < 0.05; #: *P* < 0.01; ★: *P* < 0.001

To examine cell proliferation and migration, scratch wound migration assays were conducted. Flavone, apigenin and luteolin effectively reduced the migration of MCF-7 cells into the wounded area (Fig. 3a, b). To obtain further details, cell migration was measured in real time, and treatment with flavone, apigenin, and luteolin decreased the migration ability of MCF-7 cell (Fig. 3c). These results confirm that flavone, apigenin, and luteolin directly inhibit MCF-7 cell migration, ruling out the influence of proliferation on cell motility.

### Flavone, apigenin, and luteolin activate FOXO3a, which is associated with a change in the signal transduction pathway

**Fig. 4:**
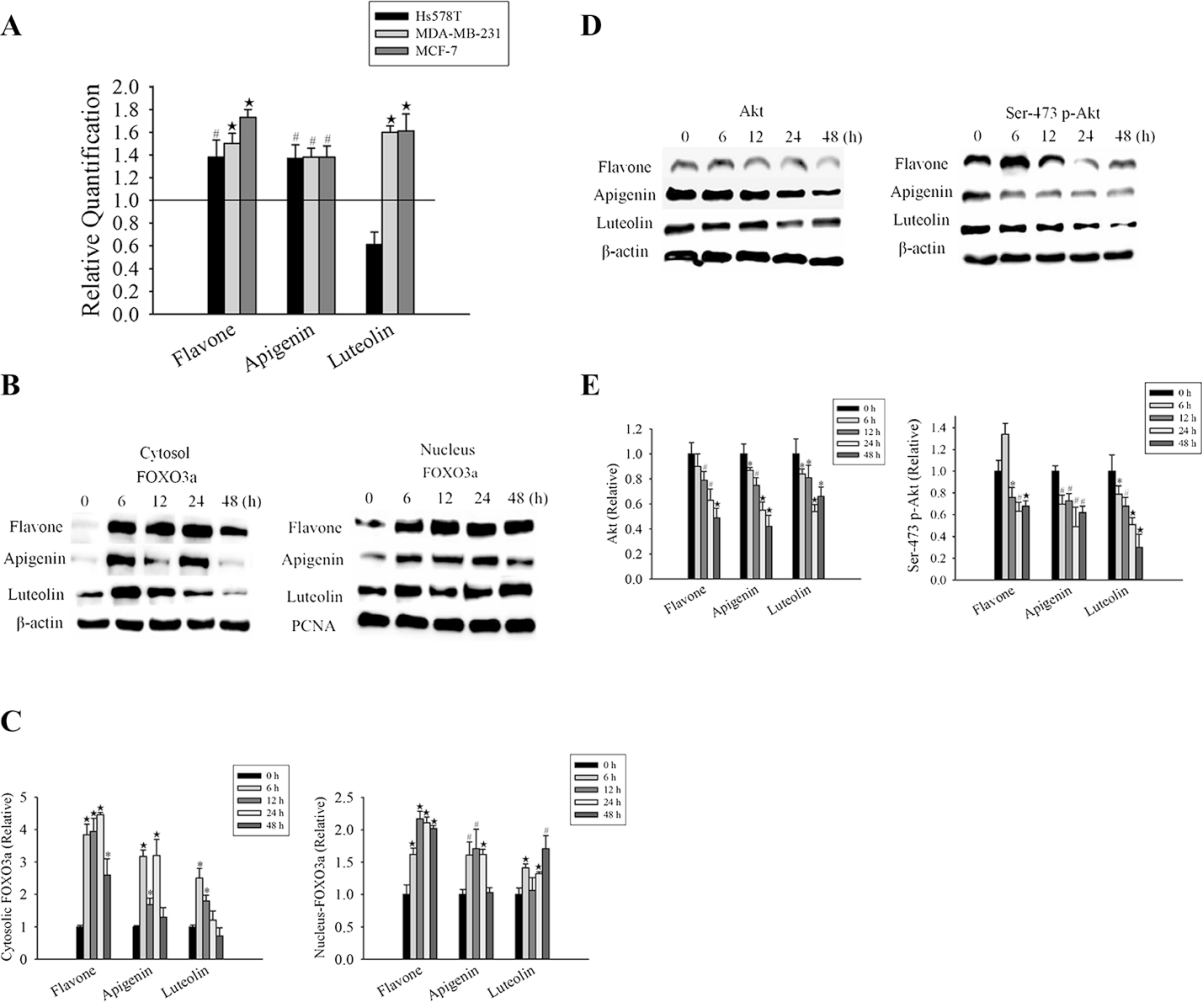
Flavone, apigenin, and luteolin activate FOXO3a, which is associated with a change in the signal transduction pathway. **a** Real-time PCR for FOXO3a. HS578T, MDA-MB-231 and MCF-7 cells were treated with the IC_50_ concentrations (Table 1) of flavone, apigenin, and luteolin for 0 h (control) and 24 h. **b** Western blot analyses of total FOXO3a in cytoplasmic and nuclear extracts isolated from MCF-7 cells treated with the IC_50_ concentrations (Table 1) of flavone, apigenin, and luteolin for various time from 0 to 48 h. Loading controls: cytoplasmic, β-actin; nuclear, proliferating cell nuclear antigen (PCNA). **c** Densitometric quantification of the FOXO3a expression from the western blot analyses. **d** MCF-7 cells were treated with IC_50_ concentrations (Table 1) of flavone, apigenin, and luteolin for 0–48 h. Western blot analyses of the total protein kinase B (Akt) and Ser-473-phosphorylated Akt (p-Akt) in cytoplasmic and nuclear extracts isolated from MCF-7 cells treated with the IC_50_ concentrations (Table 1) of flavone, apigenin, and luteolin for 0–48 h. Loading control: β-actin. **e** Densitometric quantification of the Akt and Ser-473-phosphorylated Akt expression from the western blot analyses. Results are the mean ± standard deviation of three independent experiments. *P* < 0.05 is considered as statistically significant. Symbols: *: *P* < 0.05; #: *P* < 0.01; ★: *P* < 0.001

We further determined the effect of flavone, apigenin, and luteolin on the expression of FOXO3a, a transcription factor and tumor suppressor, in the three cancer cell types. Treatment of Hs578T, MDA-MB-231, and MCF-7 cells with flavone, apigenin, and luteolin for 24 h led to an increase in the expression RNA levels of FOXO3a (Fig. 4a). To investigate whether flavone, apigenin and luteolin affect the FOXO3a expression in breast cancer cells, we performed western blot analyses on the nuclear and cytoplasmic fractions of MCF-7 cells treated with the IC_50_ concentrations of flavone, apigenin, and luteolin for 48 h. We found that these compounds increased the expression of FOXO3a in all the cells (Fig. 4b).

FOXO3a is downstream target of Akt. Akt kinase regulates breast cancer proliferation and survival [35]. Inhibiting Akt phosphorylation modulates the activities of FOXO3a and subsequently affects cell proliferation, apoptosis, and differentiation [36]. We therefore examined the roles of flavone, apigenin, and luteolin in Akt signaling. Akt was predominantly phosphorylated in control cells, whereas Akt phosphorylation in cells treated with flavone, apigenin, and luteolin for 48 h showed a marked decrease which was consistent with the decreased expression levels of FOXO3a protein (Fig. 4c, d).

**Fig. 5:**
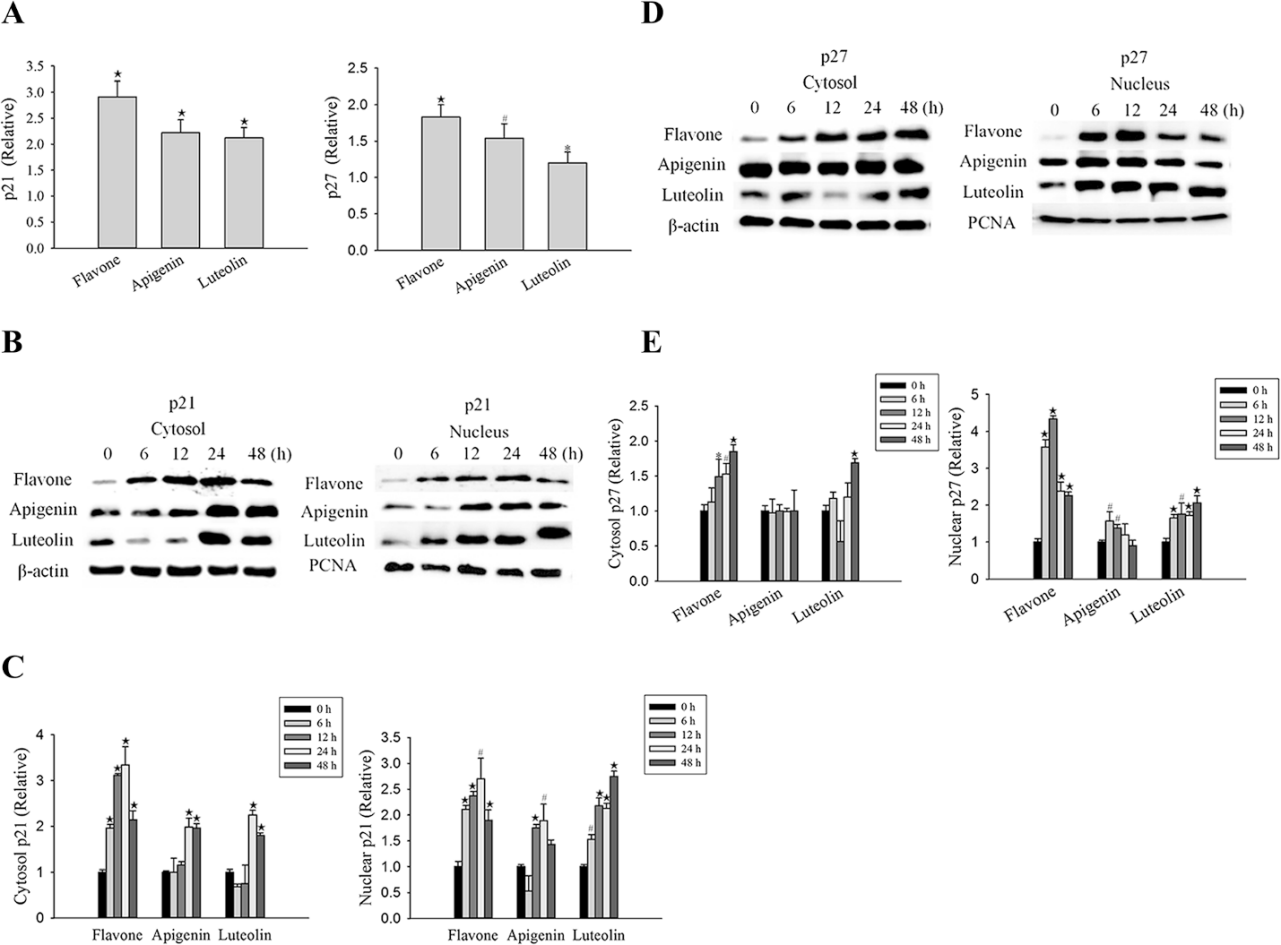
Flavone, apigenin and luteolin regulate the expression of the cyclin-dependent kinase inhibitors p21 and p27 through the Akt-FOXO3a signaling axis. **a** Real-time PCR for *p21* and *p27* expression in untreated MCF-7 cells (control) and cell treated with the IC_50_ concentrations (Table 1) of flavone, apigenin, and luteolin for 0 and 24 h. **b** Western blot analyses of total p21 in cytoplasmic and nuclear extracts isolated from MCF-7 cells treated with the IC_50_ concentrations (Table 1) of flavone, apigenin, and luteolin from 0 to 48 h. **c** Densitometric quantification of the p21 expression from the western blot analyses. **d** Western blot analyses of total p27 in cytoplasmic and nuclear extracts isolated from MCF-7 cells treated with the IC_50_ concentrations (Table 1) of flavone, apigenin and luteolin for 0–48 h. **e** Densitometric quantification of p27 expression from western blot analyses. Loading control (B and C): cytoplasmic, β-actin: nuclear proliferating cell nuclear antigen (PCNA). Results are the mean ± standard deviation of three independent experiments. *P* < 0.05 is considered as statistically significant. Symbols: *: *P* < 0.05; #: *P* < 0.01; ★: *P* < 0.001

We found that all three flavones suppressed Akt phosphorylation and increased FOXO3a expression. Akt inhibits of p21 and p27 promoter activity through reduction of FOXO3a expression [36–38]. Previous studies have suggested that anti-cancer drugs up-regulated p21 and p27, and this effect may play an important role in drug-induced cell cycle arrest in human cancer. Therefore, we examined the expression of the proteins p21 and p27, which are known targets of FOXO3a in MCF-7 cells (Fig. 5). The results indicated the flavone, apigenin, and luteolin induced upregulation of FOXO3a, which subsequently induced the expression of p21 and p27. To confirm this finding, parallel cell cultures were treated with flavone, apigenin, and luteolin, and found an increase in *p21* and *p27* mRNA levels (Fig. 5a). This finding suggests that the increased expression levels of p21 and p27 observed in the western blot analyses (Fig. 5b–d) resulted from an increase in transcription. These results are consistent with our observation of alterations in PI3K/Akt, FOXO3a, p21, and p27 expression levels after treatment with flavone, apigenin, or luteolin, suggesting that the flavone compound-mediated inhibition of cell proliferation and apoptosis were mediated at least part by regulation of the PI3K/Akt/FOXO3a/p27 signaling pathway.

## Discussion

Despite recent advances in medicinal chemistry, intrinsic and acquired resistance to chemotherapy treatments and the possibility of relapse present drawbacks in the treatment of breast cancer [39]. Because of the clear risks posed by chemotherapy, researchers worldwide have started searching for natural products that have better anti-carcinogenic activity without side effects. In addition, we believe that long chemotherapy treatment weakens the immunological defense system of the body and leaves patients susceptible to other infections and diseases. Radiation therapy is another treatment to combat cancer but also shows several potentially harmful side effects including weakened immune system and the potential to induce carcinogenesis itself. Therefore, there is an urgent need to develop chemoprevention approaches for the prevention of cancer. Chemoprevention is an important function area of oncology that focuses on the prevention of cancer using natural or synthetic agents. Recently, natural plant extracts and compounds have received widespread attention because of their potential beneficial effects on human health. Natural compounds provide a wide array of potential drug candidates for cancer therapy with various roles and targets [40, 41]. Accumulating evidence indicates that curcumin [15], quercetin [42], emodin [43], resveratrol [14] and wogonin [44] of natural origin induce apoptosis and inhibition of cell proliferation in multiple tumor cell lines including A549 lung cancer cells, hepatoma HepG2 cells, MCF-7 breast cancer cells and LNCaP prostate carcinoma cells. Flavonoids are the most common polyphenolic compounds, as they are ubiquitously present in foods of plant origin [45].

Many practitioners have used flavone compounds to treat a wide variety of ailments, including cancer [21, 23, 24, 46, 47]. Accumulating evidence indicates flavone compounds have been shown to have anti-cancer and anti-proliferative activities in *in vitro* and *in vivo* [48, 49]. In this study, we examined the mechanisms by which flavone, apigenin and luteolin induced cytotoxicity in breast cancer cells. We have shown that flavone, apigenin and luteolin induce cell cycle arrest and apoptosis in breast cancer cells. The induction of cell apoptosis occurs in response to various stresses, including activation of p53 [50], which leads to its nuclear translocation and activation of targets such as cyclin D1 and p21 that regulate the cell cycle and trigger apoptosis [51, 52]. Our results indicate that the cell cycle arrest of MCF-7 cells treated with three compounds may be associated with the inhibition of cyclin B and cyclin D1-mediated cell cycle-arrest responses. Therefore, flavone, apigenin and luteolin may inhibit breast cancer cells proliferation via cell cycle arrest and apoptosis.

The PI3K-Akt signaling pathway plays a vital role in tamoxifen and cytotoxic chemotherapeutic drug resistance [6, 7, 53], and enhanced Akt activity has been shown to elevate resistance to tamoxifen and cytotoxic drugs by promoting cell proliferation and survival [54]. Many cancers acquired drug resistance by PI3K/Akt pathway activation, which has been observed during the administration of paclitaxel in breast cancer [55]. Our results showed that flavone, apigenin and luteolin treatment substantially suppressed PI3K/Akt phosphorylation at Ser473 in MCF-7 cells.

Members of the forkhead class O (FOXO) family of transcription factors are crucial for regulating various physiological processes, including proliferation, metabolism, cell differentiation, cell cycle arrest, DNA repair and apoptosis [56]. FOXO3a are important targets of PI3K/Akt signaling pathway [11]. The Akt mediated phosphorylation of FOXO3a is known to transport FOXO3a out of nucleus and retain FOXO3a in the cytoplasm [57]. FOXO3a has also been shown to regulate cell cycle arrest and apoptosis through the activation of transcriptional targets such as p27 and p21 [36]. The nuclear localization of FOXO3a and its subsequent transcriptional activity were known to be a prognosis marker for breast cancers [9]. Our results demonstrated that treatment of breast cancer cells with flavone, apigenin, and luteolin for 12 h led to an inhibiting Akt activation and increasing the expression levels of FOXO3a, which subsequently increase the expression levels of p27 and p21 to inhibit the proliferation of breast cancer cells.

Metastasis is complex processes and accounts for the death of most cancer patients. In wound healing assay, we found that treatment of the flavone, apigenin and luteolin suppressed MCF-7 cells migration. Inhibiting Akt signaling reduced the migration and invasion of gastric cancer cells [58], which may be due to up-regulation FOXO3a. In renal cancer cells, FOXO3a has been identified as a key factor in metastasis. Overexpression of FOXO3a in renal cancer cells could inhibit tumor metastasis [59]. Flavone, apigenin and luteolin inhibit breast cancer cells migration was through inhibiting Akt activation and increasing FOXO3a expression.

**Fig. 6:**
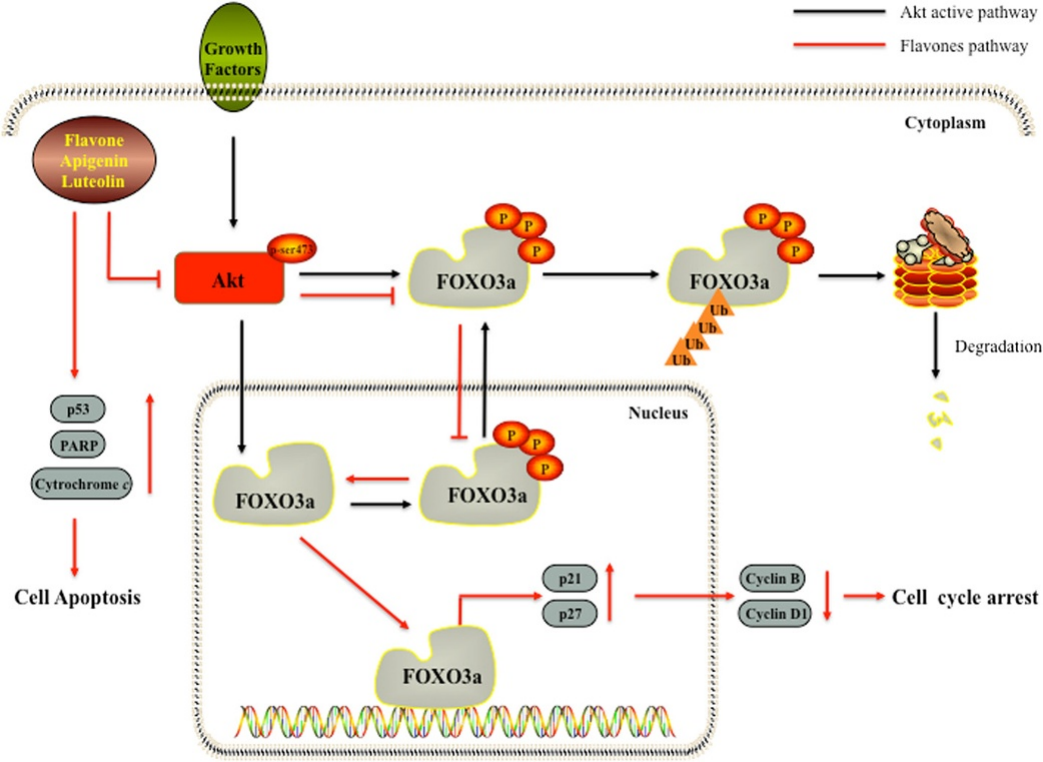
Molecular mechanism underlying the anticancer effects of flavone, apigenin, and luteolin on the Akt/FOXO3a/p27 pathway in regulating human breast cancer cell proliferation

Epidemiologic and clinical studies suggest that higher intake of plant flavonoids can prevent cancer through their interaction with various genes and enzymes [60]. Our study demonstrated that these three compounds suppressed cell proliferation in human breast cancer cells, in part, by acting on the Akt/FOXO3a/p27 signaling pathway. Flavone, apigenin, and luteolin also inhibited the proliferation of Hs578T, MDA-MB-231 and MCF-7 cells by promoting cell cycle arrest, cell apoptosis and inhibiting cell migration and invasion. These effects were associated with FOXO3a activation. These compounds may thus reduce the risk of carcinogenesis by affecting the Akt/FOXO3a/p27 signaling pathway and serve as chemopreventive agents (Fig. 6).

## Conclusions

Collectively, our study findings suggests that flavone, apigenin, and luteolin have chemopreventive properties against breast cancer, and these compounds are useful as potential preventive or therapeutic agent in the management of human breast cancer.

## Abbreviations

FOXO3a: Forkhead box O3
FOXO: Forkhead box class
Akt: Protein kinase B
PI3K: Phosphatidylinositol-3-kinase
CKI: Cyclin-dependent kinase inhibitor
PARP: (poly(ADP) polymerase
p53: Tumor protein 53
FBS: Fetal bovine serum
RT-PCR: Reverse transcription-PCR
IC50: Half maximal inhibitory concentration
M-PER: Mammalian protein extraction reagent
PVDF: Polyvinylidene difluoride
ECL: Enhanced chemiluminescence
MTT: 3-[4,5-dimethylthiazol-2-yl]-2,5diphenyl tetrazolium bromide
